# Case report: an atypical case of EBLV-1 infection in the long distance migrant bat *Pipistrellus nathusii* in mainland France

**DOI:** 10.1007/s11259-026-11166-8

**Published:** 2026-03-30

**Authors:** Evelyne Picard Meyer, Yannick Blanchard, Emmanuelle Robardet, Hervé Bourhy, Perrine Parize, Laurent Dacheux, Florence Cliquet, Véronique Beven, Edouard Hirchaud, Suzel Hurstel, Anais Pessato, Charlotte Roemer, Franck Boué, Alexandre Servat

**Affiliations:** 1 Nancy Laboratory for Rabies and Wildlife, ANSES, Malzéville, 54220 France; 2 Unit of Viral Genetics and Biosafety, Ploufragan-Plouzané-Niort Laboratory, ANSES, Ploufragan, France; 3 Lyssavirus Epidemiology and Neuropathology Unit, Institut Pasteur, Université Paris Cité, Paris, France; 4 Ligue pour la Protection des Oiseaux (LPO Alsace), 1 rue du Wisch, Rosenwiller, 67560 France; 5 Groupe d’Etude et de Protection des Mammifères d’Alsace (GEPMA), 8 rue Adèle Riton, Strasbourg, 67000 France; 6 Centre d’Ecologie et des Sciences de la Conservation (CESCO, UMR 7204), CNRS, MNHN, Sorbonne-Université, Paris, France; 7 CEFE, Univ Montpellier, CNRS, EPHE, IRD, Montpellier, France; 8 Present Address: Institut Pasteur, Université Paris Cité, Environment and Infectious Risks (ERI) Unit, Paris, France

**Keywords:** Bat rabies, Lyssavirus, EBLV-1, Nathusius’s pipistrelle, Movement, Migration

## Abstract

**Background:**

Over the past five decades, nearly 1,500 bat rabies cases have been reported in Europe. The majority of positive cases (with 90% of cases) originated from Germany, followed by The Netherlands, Denmark, Poland, France and Spain. Among the seven lyssaviruses detected in European bats, three different recognized species are circulating in some native bat species in France: EBLV-1, BBLV, and LLEBV, primarily associated with the host species *Eptesicus serotinus*, *Myotis nattereri*, and *Miniopterus schreibersii*, respectively.

**Case presentation:**

In 2022, we reported an unusual case of EBLV-1 infection in a long-distance migrant bat species, *Pipistrellus nathusii*. The bat’s brain tested positive for the presence of viral antigens using the fluorescent antibody test (FAT) and for viral RNA through molecular techniques (including real-time and end-point pan-lyssavirus RT-PCR). In this study, we determined the nearly complete genome sequence of the virus using next-generation sequencing (PX733926). Molecular clock dating and phylogenetic analysis confirmed that the bat was infected with the EBLV-1a variant, the most common and widely distributed European bat lyssavirus. The virus genome showed 99.88% nucleotide similarity with a strain isolated from a serotine bat in the Lower Saxony region of Germany in 2016 (OU524432).

**Conclusions:**

This finding is the first of its kind in France and the third in Europe involving a long-distance migrant bat. It may partially explain the extensive geographic distribution of EBLV-1a across Europe, following both east-west and north-south axes.

**Supplementary Information:**

The online version contains supplementary material available at 10.1007/s11259-026-11166-8.

## Background

Zoonotic pathogens, naturally transmitted between vertebrates, are highly diverse, encompassing viruses, bacteria, parasites, and fungi. These pathogens can spread through various transmission routes, including direct contact, inhalation, ingestion or transmission via intermediate vectors (Rahman et al. 2020). Notable examples of zoonotic viral diseases include rabies, swine and avian Influenza, or West Nile virus disease (Di Bari et al. 2023).

Migratory movements of flying vertebrates, particularly in birds (class Aves), have been clearly identified as being important for the spread of infectious agents over large spatial scales, particularly because of the vast geographic scale they encompass (Boulinier et al. 2016). Birds and bats (order Chiroptera, class Mammalia), with over 10,000 and ∼1,500 species worldwide, respectively, are recognized as natural hosts for numerous infectious viral agents (Nabi et al. 2021), such as Hendra virus, Nipah virus, and lyssaviruses in bats.

The higher proportion of zoonotic viruses in these two taxa can be attributed to several convergent features they share including high population densities, close social interactions, spatial mobility, ability to exploit diverse ecological niches, adaptability to anthropogenic environments and new habitats, elevated metabolic rates, unique immunological adaptations, and high spatial mobility that may enable them to coexist with a wide range of pathogens, over thousands of kilometers (Nabi et al. 2021).

Bats, which represent nearly one-fifth of all mammals worldwide (Burgin et al. 2018), are found across the globe except at the poles (i.e., the Arctic, Antarctic, and a few isolated oceanic islands). They are the only mammals capable of true and sustained flight. Due to their high mobility, social nature, and colonial behavior, —where some species can form aggregations of thousands of individuals— bats are ideal vectors for pathogen exchange and dispersal (Rupprecht et al. 2011).

Bats can perform various types of aerial movements for numerous reasons, including resource gathering, searching for conspecifics or suitable shelters, and avoiding predation and competition. These movements can be broadly categorized into two main types: short-distance daily journeys within the home range of a given roost (i.e. foraging areas, drinking spots, visiting new potential roosts, roosting sites) and long-distance seasonal journeys in which bats may travel long distances (i.e. to find hibernation roost, reproductive partner in swarming sites, find summer sites with lower competition for food, gather with other females at maternity colonies) (Voigt et al. 2017).

Migration, dispersal and seasonal movements are key events in the bat life cycle. The migration is defined as a seasonal, usually two-way movement between sites to escape unfavorable climatic conditions and/or to seek more favorable energetic conditions (Fleming and Eby 2003). In contrast, dispersal of bats is a one-way movement from one location to another, often from their natal site, undertaken by juveniles or immature bats (Moussy et al. 2013). While migration and dispersal are well studied in birds, they remain poorly studied in bats (Moussy et al. 2013). In Europe, several bat species can migrate long distances. Five species (*Nyctalus noctula*, *Nyctalus leisleri*, *Nyctalus lasiopterus*, *Vespertilio murinus* and *Pipistrellus nathusii*) out of the 55 European bat species are long distance migrants and are known to migrate over than 1,000 km (Vasenkov et al. 2023; Popa-Lisseanu and Voigt 2009). Coupled with the vast diversity of bats in terms of species, diet and roost sites, temperate bats exhibit also three broad patterns of seasonal movements between their summer and winter grounds and vice-versa: (i) short-distance seasonal movements (< 100 km), (ii) regional seasonal movements (100–500 km) and (iii) long-distance seasonal movements (> 500 km) (Fleming 2019). These movement behaviours are driven by several factors such as: climatic conditions, roost temperature, local food resources, the presence of suitable micro-habitats, geographical availability of roosts or the species biological cycle, particularly reproduction (Fleming and Eby 2003).

Currently, the International Committee on Taxonomy of Viruses (https://ictv.global/) recognizes 18 species among the genus *Lyssavirus*, in addition to three related, unclassified viruses. Six species are reported in insectivorous bats in Western Europe, namely *Lyssavirus bokeloh (BBLV) in Myotis nattereri* (Freuling et al. 2011); *Lyssavirus hamburg* (EBLV-1) in Serotines (*E. serotinus* and *E. isabellinus)*; *Lyssavirus helsinki* (EBLV-2) in *M. daubentonii* (McElhinney et al. 2018); *Lyssavirus caucasicus* (WCBV*)* in *Miniopterus schreibersii* (Kuzmin et al. 2005), *Lyssavirus lleida (LLEBV)* also isolated in *M. schreibersii* (Arechiga Ceballos et al. 2013) and *Lyssavirus kotalahti* (KBLV) in *M. brandtii* (Nokireki et al. 2018).

Bat rabies cases in Europe are principally attributed to EBLV-1 and EBLV-2. Nearly 1,500 rabies cases have been reported in bats across Europe between 1977 and 2025 (https://www.who-rabies-bulletin.org/). Reported for the time in Hamburg, Germany, in 1954, EBLV-1 is the most commonly found species in the continent (> 97%) with a wide distribution across Germany, The Netherlands, Denmark, France, and Poland counting for ~ 90% of all positive cases (https://www.who-rabies-bulletin.org). EBLV-2 which accounts for fewer cases (McElhinney et al. 2018), with 39 suspected cases, of which 34 confirmed by virus typing, is reported from a few counties only, including Switzerland, The Netherlands, Germany, Norway, Finland and UK (McElhinney et al. 2018). While EBLV-2 is restricted to *M. daubentonii* and *M. dasycneme*, EBLV-1 subdivided into two lineages, EBLV-1a and EBLV-1b, is primarily associated with two bat species: *E. serotinus* and *E. isabellinus*, both known for short-distance seasonal movements (https://www.iucnbsg.org/uploads/6/5/0/9/6509077/eu_bats_action_plan.pdf). The Netherlands, France, and Germany are the only countries, in which both EBLV-1a and EBLV-1b have been reported (Muller et al. 2007; Van der Poel et al. 2005; Troupin et al. 2017).

The longest movement reported for *E. isabellinus* is < 40 km, (https://www.gbif.org/species/5787585), while for *E. serotinus*, it is < 330 km (https://www.gbif.org/species/2432359). Though *E. isabellinus* (a medium-sized bat of 13–24 g with a wingspan of ~ 27.3 cm) is similar to *E. serotinus* (a larger bat weighing 18–25 g with a wingspan of ~ 37 cm), they are two morphologically cryptic but genetically distinct species with different geographical distributions. *E. isabellinus* has a fragmented distribution limited to the southern half of the Iberian Peninsula and North Africa, while *E. serotinus* has a broader distribution across Western Europe, from England to the western Iberian Peninsula and Central Asia (Juste et al. 2013).

From 1989 to 2024, 144 cases of rabies in bats were reported in mainland France with a proportion of positives of 2.1%. Three species (EBLV-1, BBLV and LLEBV) circulate among several bat species, with 139 cases of EBLV-1 associated with *E. serotinus*, one case with *P. pipistrellus* (Parize et al. 2020), two BBLV cases with *M. nattereri*, one LLEBV case with *M. schreibersii*, respectively, in addition to the positive Nathusius’ pipistrelle. We describe in this case report the discovery and genetic characterization of an EBLV-1a case in France in the long-distance migrant bat, Nathusius’ pipistrelle.

## Case presentation

### Origin and circumstances of the discovery

On May 13, 2020, an apparently weak bat was found on a terrace of a private house in Erstein (latitude 48.42740, longitude 7.66329) in eastern France. The location is in the Bas-Rhin county (area of 4,755 km²) within the Alsace region, close to Germany (air-line distance of ~ 20 km) and Switzerland (~ 90 km). The injured bat was found in the Rhine Valley, about 5 km from the Rhine river itself, in an area characterized by vast forests near the river. The Rhine, which is 1,230 km long is recognized as an important migration corridor for the Nathusius’s pipistrelle (Schwaab et al. 2009). It flows through several European countries, including France, Germany, Switzerland and the Netherlands.

On the day of its discovery, the bat was taken to a bat care center in Rosenwiller (LPO Alsace), a rehabilitation facility authorized to receive and care for injured wild animals, including bats. The bat was morphologically identified by local caretakers as a male *P. pipistrellus*. Upon arrival, it exhibited signs of exhaustion, hypothermia, and a wound on its chin. The bat’s condition deteriorated six days after admission. It died on May 19, 2020, and the carcass was kept frozen at < -18 °C.

## Laboratory analysis

### Rabies diagnostic

The bat carcass was submitted for rabies testing on January 24, 2022. Rabies testing was conducted on the brain according to the WOAH’s recommended rabies diagnosis tests using the fluorescent antibody test [FAT] (Picard-Meyer et al. 2014), conventional and real-time pan-lyssavirus SYBR-Green RT-PCR (Picard-Meyer et al. 2015) for the detection of viral antigen and RNA, respectively. Brain smears were shown positive for lyssavirus antigen by using FAT. The results were subsequently confirmed by RT-PCR detection of genomic RNA. The brain finally showed positivity with high levels of viral RNA by SYBR Green RT-PCR with the detection of ∼8 10^6 copies/µL of RNA extract.

### Genetic bat identification

We amplified the partial cytochrome b (*CytB*) gene of mitochondrial DNA (148-bp fragment of *Cytb*) after DNA extraction from a wing punch (8 mm, ∼ 0.02 mg) from the bat carcass and the partial 16S rRNA gene (560-bp) to genetically assign the bat species. Primers previously described in Lopez-Oceja et al. (Lopez-Oceja et al. 2016) were used for *CytB*: forward primer L15601: 5’-TACGCAATCCTACGATCAATTCC-3’ and reverse primer H15748: 5’-GGTTGTCCTCCAATTCATGTTAG-3’). For the amplification of the partial 16S rRNA, we used the forward (16SAr-L: 5’ -CGCCTGTTTATCAAAAACAT- 3’) and reverse (16SBr-H: 5’ -CCGGTCTGAACTCAGATCACGT- 3’) primers, previously referenced (Ruedi et al. 2023). The two amplifications were performed in a 25µL reaction volume containing 5 µL of DNA diluted to 1/10 (v/v), 2.5 µL of 10X PCR Buffer without MgCl_2_ (Invitrogen, Marseille, France), 1 µL of 50 mM MgCl_2_, 1 µL of dNTPs (10 mM each), 0.5 µL of Taq DNA polymerase (5 U) (Invitrogen, Marseille, France) and 1 µL of each forward and reverse primer (0.4 µM). The PCR was performed with the following conditions: 3 min at 95 °C, 45 cycles of 30 s at 95 °C, 60 s at 48 °C/50°C (for *CytB*/16S rRNA, respectively) and 45 s at 72 °C, following with a final step of extension of 5 min at 72 °C. PCR products were sent for Sanger sequencing (with the same PCR primers) to a commercial company (Eurofins, Ebersberg, Germany). Sequence alignment and genetic determination was carried out as previously described (Arnaout et al. 2022).

The genetic determination did not confirm the initial morphological determination of the bat as a *P. pipistrellus*, the nucleotide sequence of the *Cytb* PCR products indeed revealed more than 99% of nucleotide similarity with the species *P. nathusii* (MN122914). A second morphological determination was performed by the laboratory using the morphological criteria keys including: forearm length, length of fifth and third finger, ear and analysis of teeth under a binocular loupe and confirmed the determination of the bat species as a Nathusius’ pipistrelle. Finally, results were corroborated by the BLAST analysis of the 16 S rRNA PCR products with 100% of nucleotide similarity with the species *P. nathusii* (i.e. OQ708001.1 ; AF326104.1 ; MN122914.1).

### Viral characterization

For the extraction of RNA and hnRT-PCR, 10% (m/w) brain material suspension was prepared using PBS 1x then centrifuged at 1,500 g for 10 min. Viral RNA was extracted from 140µL clarified supernatant using a Qiagen Viral RNA mini kit according to the manufacturer’s instructions. First and second round of polymerase chain reactions were carried out with pan-lyssavirus primers as previously described giving an amplified product of 589-bp (Picard-Meyer et al. 2004).

**Fig. 1:**
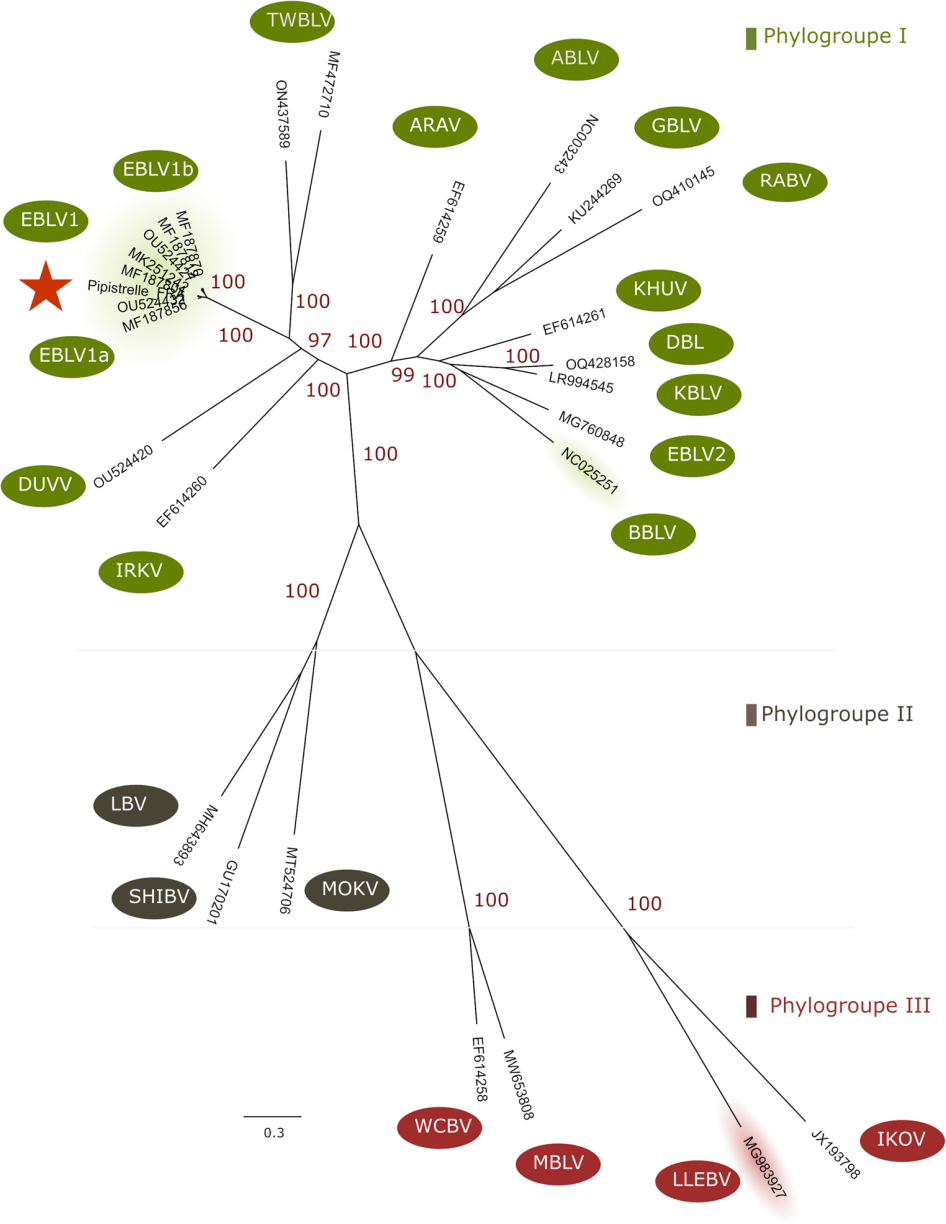
PhyML analysis of the concatenated N-P-M-G-L gene coding sequences (10,977-nt) between the Nathusius’ pipistrelle and classified lyssaviruses. PhyML methods with GTR calculation was used. Bootstrap values (100 replicates) over 70% that indicated significant support for the tree topology is shown next to the branches (in red), as well as the phylogroup (in green). ARAV: Aravan virus; ABLV: Australian bat lyssavirus; BBLV: Bokeloh bat lyssavirus; DBL: Divaea bat lyssavirus; DUVV: Duvenhage virus; EBLV-1: European bat lyssavirus 1; EBLV‐2: European bat lyssavirus 2; GBLV: Gannowura bat lyssavirus; IKOV: Ikoma lyssavirus; IRKV: Irkut virus; KBLV: Kotalahti bat lyssavirus; KHUV: Khujand virus; LBV: Lagos bat virus; LLEBV: Lleida bat lyssavirus; MBLV: Matlo bat lyssavirus; MOKV: Mokola virus; RABV: rabies virus; SHIBV: Shimoni bat virus; TWBLV: Taiwan bat lyssavirus; WCBV: West Caucasian bat virus. The Nathusius’ pipistrelle is represented with the red star

The lyssavirus identity of the isolated strain was firstly determined by Sanger sequencing on the hemi-nested RT-PCR products, then confirmed by next-generation sequencing (NGS) on the whole‐genome sequence (11963-bp) (GenBank accession number PX733926). Bio informatics analyses used were performed as previously described (Picard-Meyer et al. 2019). Comparison of the concatenated N + P+M + G+L coding sequence (10,977-nt) of the Nathusius’ pipistrelle with 7 representative sequences of EBLV-1, 18 classified lyssaviruses, one related-unclassified virus [DBLV; OQ428158] and one putative lyssavirus species [MBLV; MW653808] revealed that the Nathusius’ pipistrelle grouped perfectly with 100% bootstrap support in the phylogroup I lyssaviruses within the *Lyssavirus hamburg* species, in the subgroup formed by EBLV-1a (Fig. 1). The closest relatives were EBLV-1a from a Serotine bat identified in Germany in 2016 (OU524432), Slovenia in 2001 (MF187801) and Hungary in 2011 (MK251242). A high nucleotide similarity of 99.88% (= 11949/11963*100) was observed between lyssavirus isolates from this Nathusius’ pipistrelle and one Serotine bat from Germany (OU524432). The highest number of synonymous mutations were observed in genes coding for the phosphoprotein (*n* = 4) and the RNA polymerase (*n* = 4) followed by the glycoprotein (*n* = 1) and the matrix (*n* = 1). One synonymous substitution was also observed at the end terminus of P gene with two triplets AAA AAA instead of AAA AAC (OU524532).

### Bayesian molecular clock dating of EBLV-1

**Fig. 2:**
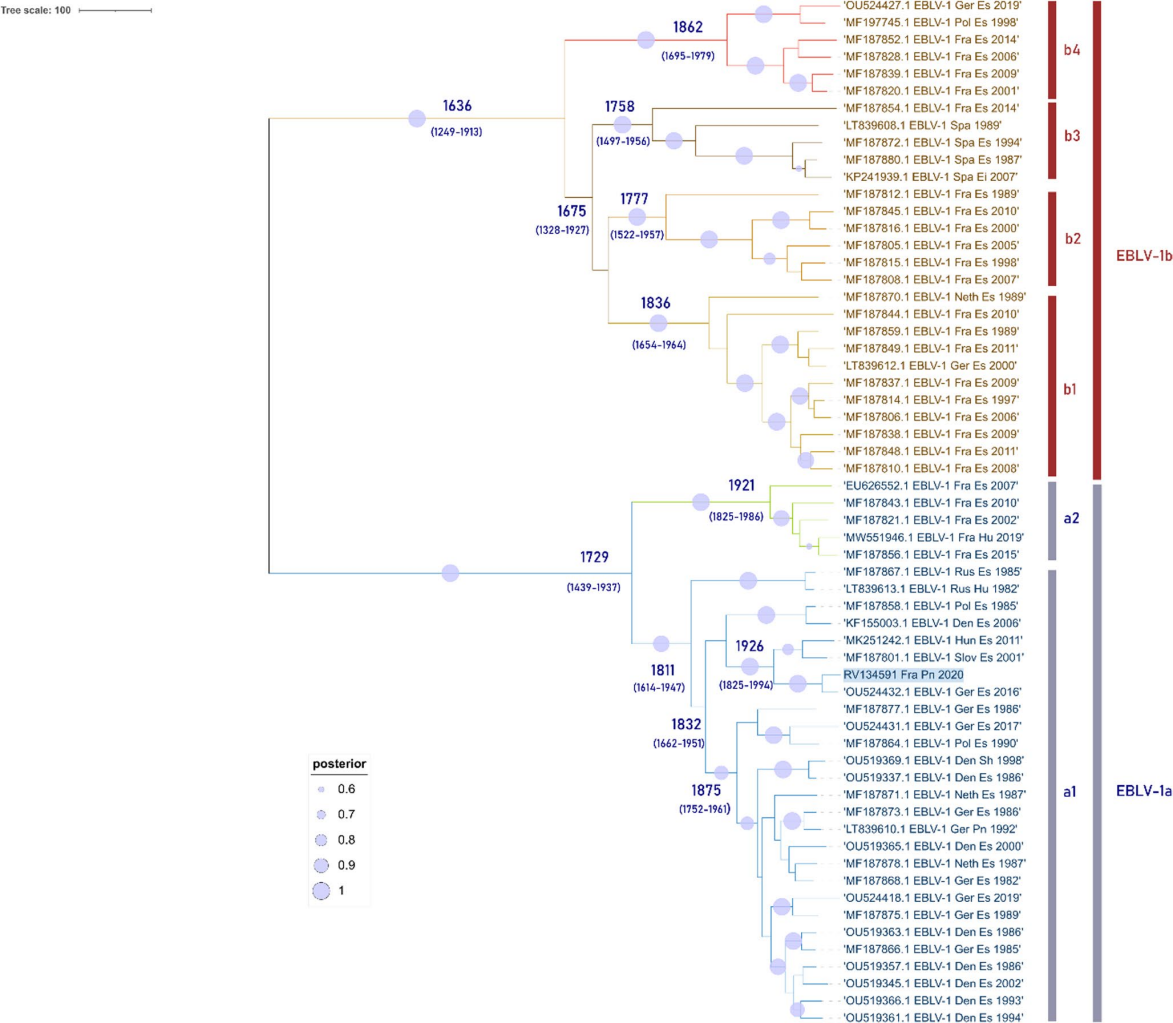
European EBLV-1 Bayesian phylogenetic reconstruction. The MCC phylogenetic tree was obtained by aligning 60 nucleoprotein gene sequences of EBLV-1 (1356-nt). The dataset of sequences included the Nathusius’ pipistrelle (numbered RV134591 FraPn 2020, highlighted in blue), 32 referenced EBLV-1a and 28 EBLV-1b sequences. Bayesian reconstruction analysis was done using the GTR substitution model and four gamma rates with a relaxed molecular clock (lognormal distribution and Bayesian skygrid model as a coalescent prior, for 50 million iterations in BEAST V (v10.5.0). The maximum clade credibility tree was annotated using Interactive Tree of Life (Itol) (v6.8.2) (Letunic and Bork 2021). Posterior probability value is given at key nodes in blue. Labels of taxa show the GenBank number accession, viral species, country, host and year of isolation

**Fig. 3:**
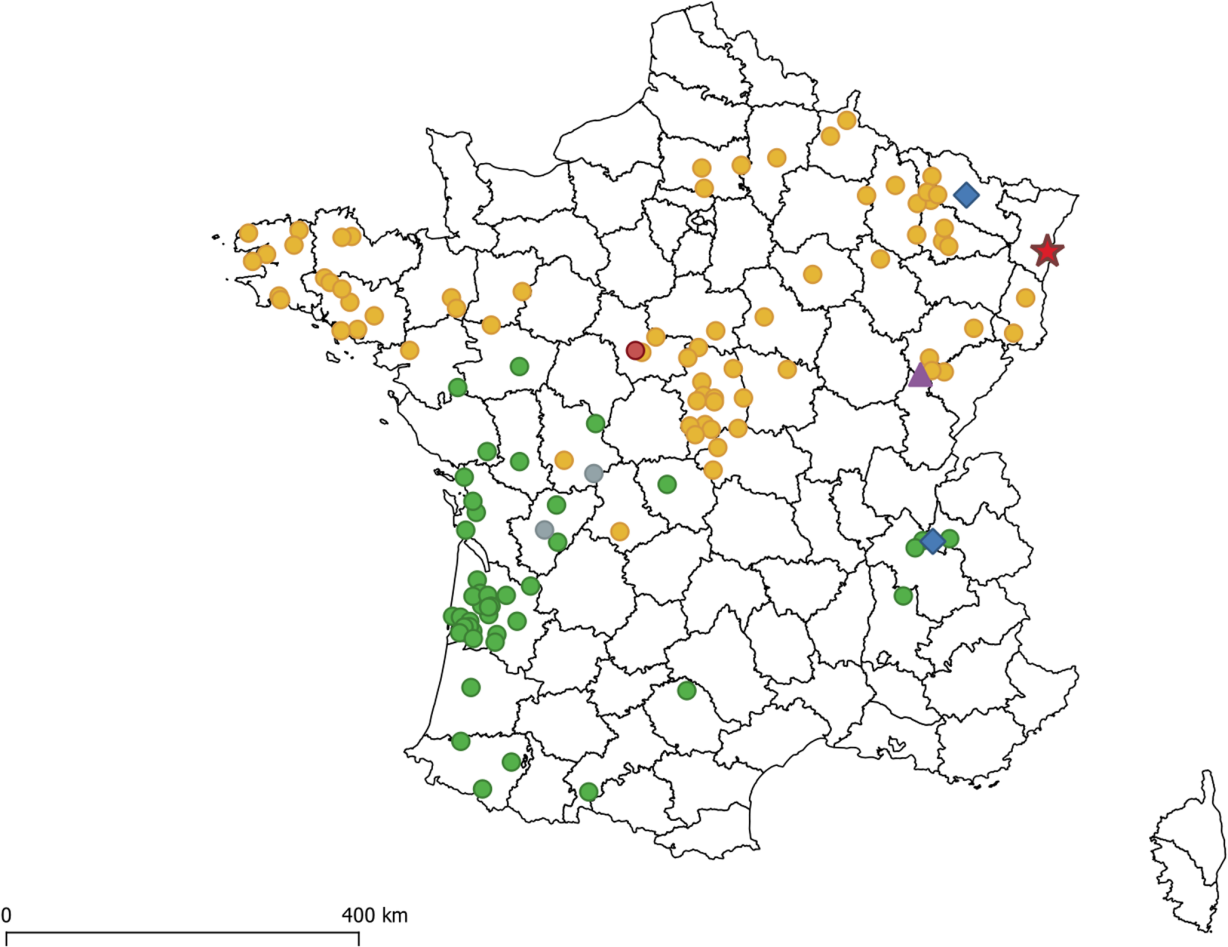
Map of France showing the location of bats infected by EBLV-1a, EBLV-1b, BBLV and LLEBV, from 1989 to 2024. Positive cases are labeled according to the identified lyssavirus species: EBLV-1a associated with *E. serotinus* (green circle), EBLV-1b associated with *E. serotinus* (orange circle), EBLV-1 associated with *E. serotinus* (gray circle), EBLV-1b associated with *P. pipistrellus* (red circle); BBLV (blue diamond), LLEBV (purple triangle). The Nathusius’ pipistrelle is represented with the red star

The time to the most recent common ancestors (TMRCA) for the two lineages EBLV-1a and b was estimated using a dataset of 60 full-length nucleoprotein gene sequences (Table S1). Bayesian Markov Chain Monte Carlo (MCMC) analyses were performed using the software BEAST X v10.5.0 and the BEAGLE library. For model parameters, we used the Yang96 option for codon partition and substitution model (GTR substitution model with four gamma rates). An uncorrelated relaxed molecular clock with a log normal distribution was used to model rate variation among branches and five MCMC chains were run for 5 × 10^7 generations each with trees and parameters sampled every 5000 steps. Analyses started with a random starting tree with a skygride model prior. Then, chains were run to an effective sample size (ESS) of parameters higher than 200 using TRACER v1.7.2. with the first 10% of trees discarded as burning. The consensus phylogenetic tree was finally built with TA then edited and annotated using iTOL (v6.8.2) (Letunic and Bork 2021). The TMRCAs of EBLV-1a and EBLV-1b have been estimated to date back to 1729 (95% HPD 1439 and 1937) and 1636 (95% HPD 1249 and 1913), respectively which is in accordance with previous results (Troupin et al. 2017). The EBLV-1a subtype in which clustered the Nathusius’ pipistrelle isolate is divided in two clusters a1, itself separated into two subgroups a1 and a2, this latter composed only by French isolates (except the Nathusius’ pipistrelle) (Fig. 2). The MCC phylogenetic tree indicates that a1 is formed by representative isolates from many European countries except for France (Denmark, Germany, Netherlands, Poland, Hungary, Slovenia, Ukraine and Russia). The cluster a1 was the first to diverge, approximately 209 years ago (95% HPD 73 and 406), while a2 formed by only French isolates, have emerged more recently, approximately 99 years ago (95% HPD 34–96) (Fig. 2). The locations of EBLV-1a and EBLV-1b cases included in the phylogenetic tree and reported in France are shown in Fig. 3.

## Discussion and conclusions

We report here an atypical association of lyssavirus EBLV-1 in Nathusius’ pipistrelle, a long-distance migrant bat found in metropolitan France. Bat lyssavirus in long distance migrants are rarely described. Among the sporadic cases of EBLV-1 spill-over in migratory bats, two cases have been reported in Nathusius’ pipistrelle, in Germany in 1986 and in 1992 respectively (King et al. 2004; Muller et al. 2007; Schatz et al. 2014a) and two cases in *Nyctalus noctula* - one in Ukraine in 1987 and one in Germany (1956–2002)- (Muller et al. 2007; Harris et al. 2006). The EBLV-1a strain confirmed in the Nathusius’ pipistrelle likely originated in Northern Europe, as it shares more than 99.8% nucleotide identity with an EBLV-1a strain (OU524432) isolated from a serotine bat in North Germany. EBLV-1 is predominantly reported in the Serotine bats, including *E. serotinus* and *E. isabellinus* (Troupin et al. 2017). Reports of sporadic detection of EBLV-1 has been made in *N. noctula*,* P. nathusii*, *P. pipistrellus*,* Rhinolophus ferrumequinum*,* Myotis myotis and Vespertilio murinus* (Muller et al. 2007; Parize et al. 2020; King et al. 2004; Selimov et al. 1991). EBLV-1 RNA has been further detected in *M. schreibersii*, *Tadarida teniotis* and *Myotis capaccinii* (Serra-Cobo et al. 2013). Limited cases have been also reported in the soprano pipistrelle bat (*P. pygmaeus*) - one case in UK and one case in Denmark (Folly et al. 2021; Calvelage et al. 2021)-, and the brown long-eared bat -one case in Germany (Schatz et al. 2014b). All these sporadic cases, which seem to indicate that EBLV-1 may circulate among various bat species, are considered as cross-species transmission events rather than independent infection cycles and remain limited.

Transmission of rabies virus usually occurs through bites or exposure of open skin or mucous membranes with infected saliva from a rabid animal. Although it is not exactly known how bat lyssaviruses spread and persist within a colony, direct physical contact through biting, scratching or licking is required for virus transmission. No clear evidence of mixed colonies-Serotine bat/Nathusius’s pipistrelle has been documented in the literature. Indeed, the two species interact with each other in roosts during migration and wintering in southwestern Europe (Brosset 1990). Both species share the same type of daytime roosts and tree holes, the backs of shutters, gaps in timber frames, piles of planks, piles of bundles, etc. are potential sites where contact can occur (Brosset 1990).

The lyssavirus carried by the Nathusius’ pipistrelle belongs to the European a1 cluster, which is clearly distinct from the a2 cluster specific to French isolates. The a1 cluster is represented by samples from at least 5 European countries - Germany, Denmark, Poland, Hungary and Slovenia. This case, which is the first of its kind in France and the third in Europe (Muller et al. 2007; Schatz et al. 2014a), raises questions about the potential role that the migratory nature of the Nathusius’s pipistrelle could play in the distribution of rabies among bats, as previously suggested (Brosset 1990),. The longest known migration distance was recorded for an individual ringed in Russia and captured in Spain, covering 2,486-km of distance (Vasenkov et al. 2022).

The bat was discovered in France in early spring, infected with a strain related to bats in northern Europe, probably during its migration from or to its summer range in northern Europe. Questions remain about the viral persistence and maintenance of EBLV-1 infection in this bat. Field studies have reported that long-distance movements in bats require substantial energy, with, for example, an investment of 25–50% of their mass in fat reserves for flight. These energetic demands and physiological trade-offs can either negatively affect their immunity and thus make these hosts more susceptible to new infections or cause reactivation of latent infections acquired during a previous season (Becker et al. 2020).

Identifying bat species using morphological keys (Dietz and Von Helversen 2004) can be challenging, for example in the case of immature or cryptic bats, and sometimes fails, which can lead to up to 12.5% of mis-identification (Arnaout et al. 2022). In this case, an inconsistency was found between the genetic and morphological determinations. These mis-identifications between *P. nathusii* and *P. pipistrellus*, also highlighted by Schlottau et al. (Schlottau et al. 2020), underscore the need to integrate genetic analysis with morphological determination, for infected bat species, in order to achieve accurate species identification, which is essential for ecological research, disease surveillance, and conservation efforts.

All lyssaviruses must be considered as potentially transmissible to humans, by bites or scratches, but effective prevention measures exist. It is easy to limit the risk of transmission and exposure by not handling, killing or capturing bats, which is prohibited in Europe, as all bats and their habitats are fully protected under the Habitat Directive (Commission of the European Communities 2003). In France, pre-exposure rabies vaccination, annual screening of rabies antibody titer, and booster vaccination doses if necessary are recommended for all bat handlers authorized to handle bats (Haut Conseil de la Santé Publique 2025), and is associated with regular and ongoing awareness-raising activities (Marmet et al. 2025). The conservation of bats is crucial for biodiversity. A total of 2.5% of them is classified as extinct or critically endangered (https://www.iucnredlist.org/). Among the multiple cases of bat mortality reported over the years, few cases have been attributed to infectious diseases of viral or bacterial origin (O’Shea et al. 2016; Dacheux et al. 2014).

To conclude, establishing a link between the ecology of bat movement, particularly in regions along migratory routes with ongoing surveillance programs, and the ecology of disease, would allow a better understanding of the role of bats in the risk of disease spread and their impact on populations (Dacheux et al. 2014; Eby et al. 2023). Questions remain about the viral persistence and maintenance of EBLV-1 infection, as well as the potential reactivation of latent infections during the long-distance movements in bats. Finally, scientifically sound communication based on results and potential risks is essential to avoid compromising decades of bat conservation efforts (López-Baucells et al. 2018).

## Supplementary Information

Below is the link to the electronic supplementary material.

Supplementary Material 1

## Data Availability

GenBank accession number PX733926 (submission of the whole-genome sequence described in the paper in the Genbank database).

## Abbreviations

BBLV: Bokeloh bat lyssavirus
CST: cross-species transmissions
CytB: cytochrome b
DBLV: Divača bat lyssavirus
EBLV-1: European bat lyssavirus 1
FAT: Fluorescent Antibody Test
KBLV: Kotalahti bat lyssavirus
LLEBV: Lleida bat lyssavirus
MBLV: Matlo bat lyssavirus
RABV: rabies virus
RT-PCR: Reverse Transcription Polymerase Chain Reaction
TMRCA: most common ancestor
WOAH: World Organisation for Animal Health

## References

[CR1] Arechiga Ceballos N, Vazquez Moron S, Berciano JM, Nicolas O, Aznar Lopez C, Juste J, Rodriguez Nevado C, Aguilar Setien A, Echevarria JE (2013) Novel lyssavirus in bat, Spain. Emerg Infect Dis 19(5):793–795. 10.3201/eid1905.121071 23648051 10.3201/eid1905.121071 PMC3647500

[CR2] Arnaout Y, Djelouadji Z, Robardet E, Cappelle J, Cliquet F, Touzalin F, Jimenez G, Hurstel S, Borel C, Picard-Meyer E (2022) Genetic identification of bat species for pathogen surveillance across France. PLoS ONE 17(1):e0261344. 10.1371/journal.pone.0261344 34982782 10.1371/journal.pone.0261344 PMC8726466

[CR3] Becker DJ, Ketterson ED, Hall RJ (2020) Reactivation of latent infections with migration shapes population-level disease dynamics. Proc Biol Sci / Royal Soc 287(1935):20201829. 10.1098/rspb.2020.1829 10.1098/rspb.2020.1829 PMC7542827 32933442

[CR4] Boulinier T, Kada S, Ponchon A, Dupraz M, Dietrich M, Gamble A, Bourret V, Duriez O, Bazire R, Tornos J, Tveraa T, Chambert T, Garnier R, McCoy KD (2016) Migration, Prospecting, Dispersal? What Host Movement Matters for Infectious Agent Circulation? Integr Comp Biol 56(2):330–342. 10.1093/icb/icw015 27252195 10.1093/icb/icw015

[CR5] Brosset A (1990) Les migrations de la pipistrelle de Nathusius, Pipistrellus nathusii, en France. Ses incidences possibles sur la propagation de la rage. Mammalia 54(2):207–212

[CR6] Burgin CJ, Colella JP, Kahn PL, Upham NS (2018) How many species of mammals are there? J Mammal 99:1–14. 10.1093/jmammal/gyx147

[CR7] Calvelage S, Tammiranta N, Nokireki T, Gadd T, Eggerbauer E, Zaeck LM, Potratz M, Wylezich C, Hoper D, Muller T, Finke S, Freuling CM (2021) Genetic and Antigenetic Characterization of the Novel Kotalahti Bat Lyssavirus (KBLV). Viruses 13(1). 10.3390/v13010069 10.3390/v13010069 PMC7825429 33419096

[CR8] Commission of the European Communities (2003) Council directive 92/43/EEC of 21 May 1992 on the conservation of natural habitats and of wild fauna and flora. https://eur-lex.europa.eu/eli/dir/1992/43/oj/eng. Accessed 17 Oct 2025

[CR9] Dacheux L, Cervantes-Gonzalez M, Guigon G, Thiberge JM, Vandenbogaert M, Maufrais C, Caro V, Bourhy H (2014) A preliminary study of viral metagenomics of French bat species in contact with humans: identification of new mammalian viruses. PLoS ONE 9(1):e87194. 10.1371/journal.pone.0087194 24489870 10.1371/journal.pone.0087194 PMC3906132

[CR10] Di Bari C, Venkateswaran N, Fastl C, Gabriël S, Grace D, Havelaar A, Huntington B, Patterson G, Rushton J, Speybroeck N, Torgerson P, Pigott D, Devleesschauwer B (2023) The global burden of neglected zoonotic diseases: Current state of evidence. One Health 17:100595. 10.1016/j.onehlt.2023.100595 37545541 10.1016/j.onehlt.2023.100595 PMC10400928

[CR11] Dietz C, Von Helversen O (2004) Illustrated identification key to the bats of Europe. Electronic version. Version 1 (2004) https://www.vleermuizenvangen.nl/images/Documenten/DietzvonHelversen2004Identificationkeybatscomplete.pdf Accessed on 10 Oct 2025.72

[CR12] Eby P, Peel AJ, Hoegh A, Madden W, Giles JR, Hudson PJ, Plowright RK (2023) Pathogen spillover driven by rapid changes in bat ecology. Nature 613(7943):340–344. 10.1038/s41586-022-05506-2 36384167 10.1038/s41586-022-05506-2 PMC9768785

[CR13] Fleming TH (2019) Bat migration. Encyclopedia Anim Behav 605–610. 10.1016/B978-0-12-809633-8.20764-4

[CR14] Fleming TH, Eby P (2003) Ecology of bat migration. In: In: Kunz T.H. FMBeBETUoCP, Chicago, Illinois, USA, pp 156–208 (ed)

[CR15] Folly AJ, Marston DA, Golding M, Shukla S, Wilkie R, Lean FZX, Nunez A, Worledge L, Aegerter J, Banyard AC, Fooks AR, Johnson N, McElhinney LM (2021) Incursion of European Bat Lyssavirus 1 (EBLV-1) in Serotine Bats in the United Kingdom. Viruses 13(10). 10.3390/v13101979 10.3390/v13101979 PMC8536961 34696409

[CR16] Freuling CM, Beer M, Conraths FJ, Finke S, Hoffmann B, Keller B, Kliemt J, Mettenleiter TC, Muhlbach E, Teifke JP, Wohlsein P, Muller T (2011) Novel lyssavirus in Natterer’s bat. Ger Emerg Infect Dis 17(8):1519–1522. 10.3201/eid1708.110201 10.3201/eid1708.110201 PMC3381583 21801640

[CR17] Harris SL, Brookes SM, Jones G, Hutson AM, Racey PA, Aegerter J, Smith GC, McElhinney LM, Fooks AR (2006) European bat lyssaviruses: Distribution, prevalence and implications for conservation. Biol Conserv 131(2):193–210. 10.1016/j.biocon.2006.04.006 32226078 10.1016/j.biocon.2006.04.006 PMC7096730

[CR18] Haut Conseil de la Santé Publique (2025) Vaccinations contre la rage et prophylaxie post-exposition. Recommandations. https://www.hcsp.fr/explore.cgi/avisrapportsdomaine?clefr=317. Accessed 07 Oct 2025

[CR19] Juste J, Benda P, Garcia-Mudarra JL, Ibanez C (2013) Phylogeny and systematics of Old World serotine bats (genus Eptesicus, Vespertilionidae, Chiroptera): an integrative approach. Zoolog Scr 42(5):441–457. 10.1111/zsc.12020

[CR20] King AA, Haagsma J, Kappeler A (2004) Lyssavirus infections in European bats. In: King AA, FAR, Aubert M, Wandeler AI (eds) Historical perspective of Rabies in Europe and the Mediterranean Basin. OIE (World organisation for animal health), Paris, pp 221–241

[CR21] Kuzmin IV, Hughes GJ, Botvinkin AD, Orciari LA, Rupprecht CE (2005) Phylogenetic relationships of Irkut and West Caucasian bat viruses within the Lyssavirus genus and suggested quantitative criteria based on the N gene sequence for lyssavirus genotype definition. Virus Res 111(1):28–43. 10.1016/j.virusres.2005.03.008 15896400 10.1016/j.virusres.2005.03.008

[CR22] Letunic I, Bork P (2021) Interactive Tree Of Life (iTOL) v5: an online tool for phylogenetic tree display and annotation. Nucleic Acids Res 49(W1):W293–W296. 10.1093/nar/gkab301 33885785 10.1093/nar/gkab301 PMC8265157

[CR23] López-Baucells A, Rocha R, Fernández-Llamazares Á (2018) When bats go viral: negative framings in virological research imperil bat conservation. Mammal Rev 48(1):62–66. 10.1111/mam.12110

[CR24] Lopez-Oceja A, Gamarra D, Borragan S, Jimenez-Moreno S, de Pancorbo MM (2016) New cyt b gene universal primer set for forensic analysis. Forensic Sci Int Genet 23:159–165. 10.1016/j.fsigen.2016.05.001 27206224 10.1016/j.fsigen.2016.05.001

[CR25] Marmet J, Dacheux L, Worsley-Tonks K, Picard-Meyer E, Bourhy H, Parize P (2025) Bat rabies exposures and safety practices among a self-selecting sample of French bat handlers. IJID One Health 8:100079. 10.1016/j.ijidoh.2025.100079

[CR26] McElhinney LM, Marston DA, Wise EL, Freuling CM, Bourhy H, Zanoni R, Moldal T, Kooi EA, Neubauer-Juric A, Nokireki T, Muller T, Fooks AR (2018) Molecular Epidemiology and Evolution of European Bat Lyssavirus 2. Int J Mol Sci 19(1). 10.3390/ijms19010156 10.3390/ijms19010156 PMC5796105 29303971

[CR27] Moussy C, Hosken DJ, Mathews F, Smith GC, Aegerter J, Bearhop S (2013) Migration and dispersal patterns of bats and their influence on genetic structure. Mammal Rev 43:183–195. 10.1111/j.1365-2907.2012.00218.x

[CR28] Muller T, Johnson N, Freuling CM, Fooks AR, Selhorst T, Vos A (2007) Epidemiology of bat rabies in Germany. Arch Virol 152(2):273–288. 10.1007/s00705-006-0853-5 17066249 10.1007/s00705-006-0853-5

[CR29] Nabi G, Wang Y, Lü L, Jiang C, Ahmad S, Wu Y, Li D (2021) Bats and birds as viral reservoirs: A physiological and ecological perspective. Sci Total Environ 754:1–6. 10.1016/j.scitotenv.2020.142372 10.1016/j.scitotenv.2020.142372 PMC7505891 33254850

[CR30] Nokireki T, Tammiranta N, Kokkonen UM, Kantala T, Gadd T (2018) Tentative novel lyssavirus in a bat in Finland. Transbound Emerg Dis 65(3):593–596. 10.1111/tbed.12833 29446230 10.1111/tbed.12833

[CR31] O’Shea TJ, Cryan PM, Hayman DTS, Plowright RK, Streicker DG (2016) Multiple mortality events in bats: a global review. Mamm Rev 46(3):175–190. 10.1111/mam.12064 29755179 10.1111/mam.12064 PMC5942905

[CR32] Parize P, Travecedo Robledo IC, Cervantes-Gonzalez M, Kergoat L, Larrous F, Serra-Cobo J, Dacheux L, Bourhy H (2020) Circumstances of Human-Bat interactions and risk of lyssavirus transmission in metropolitan France. Zoonoses Public Health 67(7):774–784. 10.1111/zph.12747 32770828 10.1111/zph.12747

[CR34] Picard-Meyer E, Bruyere V, Barrat J, Tissot E, Barrat MJ, Cliquet F (2004) Development of a hemi-nested RT-PCR method for the specific determination of European Bat Lyssavirus 1. Comparison with other rabies diagnostic methods. Vaccine 22(15–16):1921–1929. 10.1016/j.vaccine.2003.11.015 15121304 10.1016/j.vaccine.2003.11.015

[CR36] Picard-Meyer E, Robardet E, Arthur L, Larcher G, Harbusch C, Servat A, Cliquet F (2014) Bat rabies in France: a 24-year retrospective epidemiological study. PLoS ONE 9(6):e98622. 10.1371/journal.pone.0098622 24892287 10.1371/journal.pone.0098622 PMC4044004

[CR35] Picard-Meyer E, Peytavin de Garam C, Schereffer JL, Marchal C, Robardet E, Cliquet F (2015) Cross-platform evaluation of commercial real-time SYBR green RT-PCR kits for sensitive and rapid detection of European bat Lyssavirus type 1. Biomed Res Int 2015:839518. 10.1155/2015/839518 25785274 10.1155/2015/839518 PMC4345247

[CR33] Picard-Meyer E, Beven V, Hirchaud E, Guillaume C, Larcher G, Robardet E, Servat A, Blanchard Y, Cliquet F (2019) Lleida Bat Lyssavirus isolation in Miniopterus schreibersii in France. Zoonoses Public Health 66(2):254–258. 10.1111/zph.12535 30460779 10.1111/zph.12535

[CR37] Popa-Lisseanu AG, Voigt CC (2009) Bats on the Move. J Mammal 90(6):1283–1289. 10.1644/09-MAMM-S-130R2.1

[CR38] Rahman MT, Sobur MA, Islam MS, Ievy S, Hossain MJ, El Zowalaty ME, Rahman AT, Ashour HM (2020) Zoonotic Diseases: Etiology, Impact, and Control. Microorganisms 8(9). 10.3390/microorganisms8091405 10.3390/microorganisms8091405 PMC7563794 32932606

[CR39] Ruedi M, Manzinalli J, Dietrich A, Vinciguerra L (2023) Shortcomings of DNA barcodes: a perspective from the mammal fauna of Switzerland. Hystrix Italian J Mammalogy 34(1):54–61. 10.4404/hystrix-00628-2023

[CR40] Rupprecht CE, Turmelle A, Kuzmin IV (2011) A perspective on lyssavirus emergence and perpetuation. Current opinion in virology 1. 6662–670. 10.1016/j.coviro.2011.10.014 10.1016/j.coviro.2011.10.014 22440925

[CR41] Schatz J, Freuling CM, Auer E, Goharriz H, Harbusch C, Johnson N, Kaipf I, Mettenleiter TC, Muhldorfer K, Muhle RU, Ohlendorf B, Pott-Dorfer B, Pruger J, Ali HS, Stiefel D, Teubner J, Ulrich RG, Wibbelt G, Muller T (2014a) Enhanced passive bat rabies surveillance in indigenous bat species from Germany–a retrospective study. PLoS Negl Trop Dis 8(5):e2835. 10.1371/journal.pntd.0002835 24784117 10.1371/journal.pntd.0002835 PMC4006713

[CR42] Schatz J, Teifke JP, Mettenleiter TC, Aue A, Stiefel D, Muller T, Freuling CM (2014b) Lyssavirus distribution in naturally infected bats from Germany. Vet Microbiol 169(1–2):33–41. 10.1016/j.vetmic.2013.12.004 24440375 10.1016/j.vetmic.2013.12.004

[CR43] Schlottau K, Eggerbauer E, Freuling CM, Beer M, Muller T, Hoffmann B (2020) Rapid molecular species identification of indigenous bats from Germany for surveillance purposes. Infect Genet evolution: J Mol Epidemiol evolutionary Genet Infect Dis 78:104140. 10.1016/j.meegid.2019.104140 10.1016/j.meegid.2019.104140 31837485

[CR44] Schwaab F, Knochel A, Jouan D (2009) Connaître et protéger les chauves-souris de Lorraine. CPEPESC Lorraine

[CR45] Selimov MA, Smekhov AM, Antonova LA, Shablovskaya EA, King AA, Kulikova LG (1991) New strains of rabies-related viruses isolated from bats in the Ukraine. Acta Virol 35(3):226–231 1683127

[CR46] Serra-Cobo J, Lopez-Roig M, Segui M, Sanchez LP, Nadal J, Borras M, Lavenir R, Bourhy H (2013) Ecological factors associated with European bat lyssavirus seroprevalence in spanish bats. PLoS ONE 8(5):e64467. 10.1371/journal.pone.0064467 23700480 10.1371/journal.pone.0064467 PMC3659107

[CR47] Troupin C, Picard-Meyer E, Dellicour S, Casademont I, Kergoat L, Lepelletier A, Dacheux L, Baele G, Monchatre-Leroy E, Cliquet F, Lemey P, Bourhy H (2017) Host Genetic Variation Does Not Determine Spatio-Temporal Patterns of European Bat 1 Lyssavirus. Genome Biol Evol 9(11):3202–3213. 10.1093/gbe/evx236 29165566 10.1093/gbe/evx236 PMC5721339

[CR48] Van der Poel WH, Van der Heide R, Verstraten ER, Takumi K, Lina PH, Kramps JA (2005) European bat lyssaviruses, The Netherlands. Emerg Infect Dis 11(12):1854–1859 16485470 10.3201/eid1112.041200 PMC3367619

[CR49] Vasenkov D, Desmet J, Popov I, Sidorchuk NV (2022) Bats can migrate farther than it was previously known: a new longest migration record by Nathusius’ pipistrelle Pipistrellus nathusii (Chiroptera: Vespertilionidae). Mammalia 86:524–526. 10.1515/mammalia-2021-0139

[CR50] Vasenkov DA, Vasiliev NS, Sidorchuk NV, Rozhnov VV (2023) Autumn Migration of Greater Noctule Bat (Nyctalus Lasiopterus): through Countries and over Mountains to a New Migration Flight Record in Bats. Dokl Biol Sci 513(1):395–399. 10.1134/S0012496623700746 37950812 10.1134/S0012496623700746 PMC10811110

[CR51] Voigt CC, Frick WF, Holderied MW, Holland R, Kerth G, Mello MAR, Plowright RK, Swartz S, Yovel Y (2017) Principles and Patterns of Bat Movements: From Aerodynamics to Ecology. Q Rev Biol 92(3):267–287. 10.1086/693847 29861509 10.1086/693847 PMC5983048

