# Supplementary material for: Case report: an atypical case of EBLV-1 infection in the long distance migrant bat Pipistrellus nathusii in mainland France
Source: Vet Res Commun. 2026 Mar 30;50(3):240. doi: 10.1007/s11259-026-11166-8 (PMC13035623; doi:10.1007/s11259-026-11166-8)
Supplement: Supplementary file 1 — Supplementary Material 1 [file 11259_2026_11166_MOESM1_ESM.docx]

**Table S1. Details of EBLV-1 viruses used in the Bayesian MCC phylogenetic reconstruction**

| **ID Sequence** | **Date of isolation** | **Host** | **Country of isolation** | **Phylo-genetic sub-clade** | **Accession number/** | **Reference** |
| --- | --- | --- | --- | --- | --- | --- |
| **EBLV-1a :** |  |  |  |  |  |  |
| OU519337.1_EBLV-1_Den_Es_1986 | 1986 | Es | Denmark | A1 | OU519337.1 | IVD NGS Lab, 2021 |
| OU519363.1_EBLV-1_Den_Es_1986 | 1986 | Es | Denmark | A1 | OU519363.1 | IVD NGS Lab, 2021 |
| OU519357.1_EBLV-1_Den_Es_1986 | 1986 | Es | Denmark | A1 | OU519357.1 | IVD NGS Lab, 2021 |
| OU519366.1_EBLV-1_Den_Es_1993 | 1993 | Es | Denmark | A1 | OU519366.1 | IVD NGS Lab, 2021 |
| OU519361.1_EBLV-1_Den_Es_1994 | 1994 | Es | Denmark | A1 | OU519361.1 | IVD NGS Lab, 2021 |
| OU519369.1_EBLV-1_Den_Sh_1998 | 1998 | Sh | Denmark | A1 | OU519369.1 | IVD NGS Lab, 2021 |
| OU519365.1_EBLV-1_Den_Es_2000 | 2000 | Es | Denmark | A1 | OU519365.1 | IVD NGS Lab, 2021 |
| OU519345.1_EBLV-1_Den_Es_2002 | 2002 | Es | Denmark | A1 | OU519345.1 | IVD NGS Lab, 2021 |
| KF155003.1_EBLV-1_Den_Es_2006 | 2006 | Es | Denmark | A1 | KF155003.1 | Marston et al., 2013 |
| RV134591_Fra_Pn_  2020 | 2020 | Pn | France | A1 | PX733926 | This study |
| MF187821.1_EBLV-1_Fra_Es_2002 | 2002 | Es | France | A2 | MF187821.1 | Troupin et al., 2017 |
| EU626552.1_EBLV-1_Fra_Es_2007 | 2007 | Cat | France | A2 | EU626552.1 | Dacheux et al., 2009 |
| MF187843.1_EBLV-1_Fra_Es_2010 | 2010 | Es | France | A2 | MF187843.1 | Troupin et al., 2017 |
| MF187856.1_EBLV-1_Fra_Es_2015 | 2015 | Es | France | A2 | MF187856.1 | Troupin et al., 2017 |
| MW551946.1_EBLV-1_Fra_Hu_2019 | 2019 | Hu | France | A2 | MW551946.1 | Regnault et al., 2021 |
| MF187868.1_EBLV-1_Ger_Es_1982 | 1982 | Es | Germany | A1 | MF187868.1 | Troupin et al., 2017 |
| MF187866.1_EBLV-1_Ger_Es_1985 | 1985 | Es | Germany | A1 | MF187866.1 | Troupin et al., 2017 |
| MF187877.1_EBLV-1_Ger_Es_1986 | 1986 | Es | Germany | A1 | MF187877.1 | Troupin et al., 2017 |
| MF187873.1_EBLV-1_Ger_Es_1986 | 1986 | Es | Germany | A1 | MF187873.1 | Troupin et al., 2017 |
| MF187875.1_EBLV-1_Ger_Es_1989 | 1989 | Es | Germany | A1 | MF187875.1 | Troupin et al., 2017 |
| LT839610.1_EBLV-1_Ger_Pn_1992 | 1992 | Pn | Germany | A1 | LT839610.1 | Hoeper D., 2018 |
| OU524432.1_EBLV-1_Ger_Es_2016 | 2016 | Es | Germany | A1 | OU524432.1 | IVD NGS Lab, 2021 |
| OU524431.1_EBLV-1_Ger_Es_2017 | 2017 | Es | Germany | A1 | OU524431.1 | IVD NGS Lab, 2021 |
| OU524418.1_EBLV-1_Ger_Es_2019 | 2019 | Es | Germany | A1 | OU524418.1 | IVD NGS Lab, 2021 |
| MK251242.1_EBLV-1_Hun_Es_2011 | 2011 | Es | Hungary | A1 | MK251242.1 | Banyai K. and Forro B., 2019 |
| MF187858.1_EBLV-1_Pol_Es_1985 | 1985 | Es | Poland | A1 | MF187858.1 | Troupin et al., 2017 |
| MF187864.1_EBLV-1_Pol_Es_1990 | 1990 | Es | Poland | A1 | MF187864.1 | Troupin et al., 2017 |
| MF187801.1_EBLV-1_Slov_Es_2001 | 2001 | Es | Slovenia | A1 | MF187801.1 | Troupin et al., 2017 |
| MF187871.1_EBLV-1_Neth_Es_1987 | 1987 | Es | The Netherlands | A1 | MF187871.1 | Troupin et al., 2017 |
| MF187878.1_EBLV-1_Neth_Es_1987 | 1987 | Es | The Netherlands | A1 | MF187878.1 | Troupin et al., 2017 |
| LT839613.1_EBLV-1_Rus_Hu_1982 | 1982 | Hu | Russia | A1 | LT839613.1 | Hoeper D. 2018 |
| MF187867.1_EBLV-1_Rus_Es_1985 | 1985 | Es | Russia | A1 | MF187867.1 | Troupin et al., 2017 |
| **EBLV-1b :** |  |  |  |  |  |  |
| LT839612.1_EBLV-1_Ger_Es_2000 | 2000 | Es | Germany | B1 | LT839612.1 | Hoeper D. 2018 |
| OU524427.1_EBLV-1_Ger_Es_2019 | 2019 | Es | Germany | B4 | OU524427.1 | IVD NGS Lab, 2021 |
| MF187859.1_EBLV-1_Fra_Es_1989 | 1989 | Es | France | B1 | MF187859.1 | Troupin et al., 2017 |
| MF187814.1_EBLV-1_Fra_Es_1997 | 1997 | Es | France | B1 | MF187814.1 | Troupin et al., 2017 |
| MF187806.1_EBLV-1_Fra_Es_2006 | 2006 | Es | France | B1 | MF187806.1 | Troupin et al., 2017 |
| MF187810.1_EBLV-1_Fra_Es_2008 | 2008 | Es | France | B1 | MF187810.1 | Troupin et al., 2017 |
| MF187838.1_EBLV-1_Fra_Es_2009 | 2009 | Es | France | B1 | MF187838.1 | Troupin et al., 2017 |
| MF187837.1_EBLV-1_Fra_Es_2009 | 2009 | Es | France | B1 | MF187837.1 | Troupin et al., 2017 |
| MF187844.1_EBLV-1_Fra_Es_2010 | 2010 | Es | France | B1 | MF187844.1 | Troupin et al., 2017 |
| MF187849.1_EBLV-1_Fra_Es_2011 | 2011 | Es | France | B1 | MF187849.1 | Troupin et al., 2017 |
| MF187848.1_EBLV-1_Fra_Es_2011 | 2011 | Es | France | B1 | MF187848.1 | Troupin et al., 2017 |
| MF187812.1_EBLV-1_Fra_Es_1989 | 1989 | Es | France | B2 | MF187812.1 | Troupin et al., 2017 |
| MF187815.1_EBLV-1_Fra_Es_1998 | 1998 | Es | France | B2 | MF187815.1 | Troupin et al., 2017 |
| MF187816.1_EBLV-1_Fra_Es_2000 | 2000 | Es | France | B2 | MF187816.1 | Troupin et al., 2017 |
| MF187805.1_EBLV-1_Fra_Es_2005 | 2005 | Es | France | B2 | MF187805.1 | Troupin et al., 2017 |
| MF187808.1_EBLV-1_Fra_Es_2007 | 2007 | Es | France | B2 | MF187808.1 | Troupin et al., 2017 |
| MF187845.1_EBLV-1_Fra_Es_2010 | 2010 | Es | France | B2 | MF187845.1 | Troupin et al., 2017 |
| MF187854.1_EBLV-1_Fra_Es_2014 | 2014 | Es | France | B3 | MF187854.1 | Troupin et al., 2017 |
| MF187820.1_EBLV-1_Fra_Es_2001 | 2001 | Es | France | B4 | MF187820.1 | Troupin et al., 2017 |
| MF187828.1_EBLV-1_Fra_Es_2006 | 2006 | Es | France | B4 | MF187828.1 | Troupin et al., 2017 |
| MF187839.1_EBLV-1_Fra_Es_2009 | 2009 | Es | France | B4 | MF187839.1 | Troupin et al., 2017 |
| MF187852.1_EBLV-1_Fra_Es_2014 | 2014 | Es | France | B4 | MF187852.1 | Troupin et al., 2017 |

Es: *Eptesicus serotinus*; Hu: Human; Pn: *Pipistrellus nathusii* ; Sh : sheep
